# The Impact of the COVID-19 Outbreak on Patients’ Adherence to PCSK9 Inhibitors Therapy

**DOI:** 10.3390/jcm11030475

**Published:** 2022-01-18

**Authors:** Valentina Maria Caso, Simona Sperlongano, Biagio Liccardo, Emanuele Romeo, Sergio Padula, Fortunato Arenga, Antonello D’Andrea, Pio Caso, Paolo Golino, Gerardo Nigro, Vincenzo Russo

**Affiliations:** 1 Cardiology Unit, Department of Traslational Medical Sciences, University of Campania Luigi Vanvitelli, 80131 Naples, Italy; valepica8@yahoo.it (V.M.C.); sperlongano.simona@gmail.com (S.S.); liccardob@gmail.com (B.L.); ema.romeo@virgilio.it (E.R.); paolo.golino@unicampania.it (P.G.); gerardo.nigro@unicampania.it (G.N.); 2 Division of Cardiology, Monaldi Hospital, 80131 Naples, Italy; padulas@libero.it (S.P.); fortunatoarenga@libero.it (F.A.); pio.caso@ospedalideicolli.it (P.C.); 3 Department of Cardiology and Intensive Coronary Care, Umberto I Hospital, 84014 Nocera Inferiore, Italy; antonellodandrea@libero.it

**Keywords:** COVID-19, PCSK9 inhibitors, adherence, lockdown

## Abstract

PCSK9 inhibitors (PCSK9i) are monoclonal antibodies that have been shown to be effective in reducing both LDL cholesterol (LDL-C) values and major cardiovascular events in patients at high cardiovascular risk. Adherence to PCSK9i is critical for the success of the treatment. The aim of the present study is to evaluate patients’ adherence to PCSK9i during the COVID-19 pandemic. Patients referred to the Cardiac Diagnostic Unit of the University of Campania “Luigi Vanvitelli” Naples, taking PCSK9i, and who missed the cardiological follow-up visit during the first national COVID-19 lockdown (9 March–17 May 2020), were included. Each patient underwent medical teleconsultation to collect current clinical conditions, adherence to drug treatments, and lipid profile laboratory tests. Among 151 eligible patients, 20 were excluded for missing or untraceable telephone numbers and one for refusing to join the interview. The selected study population consisted of 130 patients (64 ± 9 years, 68% males), of whom 11 (8.5%) reported a temporary interruption of the PCSK-9 therapy for a mean period of 65 ± 1.5 days. The non-adherent patients showed a marked increase in LDL-C than in the pre-pandemic period (90.8 ± 6.0 vs. 54.4 ± 7.7 mg/dL, *p* < 0.0001), and 82% of patients moved out of the LDL-C therapeutic range. The non-adherent group was more likely to have a very high cardiovascular risk compared to the adherent group (81.8 vs. 33.6%, *p* < 0.001). Causes of interruption included drug prescription failure (63.6%) due to temporary interruption of the non-urgent outpatient visits and failure in drug withdrawal (36.4%) due to patients’ fear of becoming infected during the pandemic. The COVID-19 lockdown caused a remarkable lack of adherence to PCSK9i therapy, risking negative implications for the health status of patients at high cardiovascular risk. Facilitating patients’ access to PCSK9i and enhancing telemedicine seem to be effective strategies to ensure the continuity of care and appropriate management of these patients.

## 1. Introduction

The coronavirus disease 2019 (COVID-19) pandemic is a global health emergency caused by the severe acute respiratory syndrome coronavirus 2 (SARS-CoV-2) [1]. The clinical course of the disease can be asymptomatic or complicated by the onset of severe respiratory distress syndrome and/or multi-organ failure that may require hospitalization [2,3]. In attempting to contain the virus spread, the Italian Government adopted strict rules, including the national lockdown from 9 March 2020 to 17 May 2020 [4]. During this period, non-urgent clinic visits or cardiac interventional procedures were postponed to a later date and changes in the patterns of hospital admissions in Italy were observed [5,6,7,8]. To date, no data have been provided on the adherence to cardiovascular (CV) pharmacotherapy during the COVID-19 outbreak.

Among the CV therapies, proprotein convertase subtilisin/kexin 9 inhibitors (PCSK9i) are a new class of lipid-lowering drugs, which proved to be effective in reducing low-density lipoprotein cholesterol (LDL-C) and CV events in high-risk patients [9,10]. PCSK9i have a convenient administration regimen (subcutaneously, bi-weekly, or monthly) and a favorable adverse effects profile, with no risk of new-onset diabetes or cognitive deficits [9,10,11,12]. For these reasons, they are currently recommended, alone or in combination therapy with high-intensity statin and/or ezetimibe, for patients at high and very high CV risk [13]. These patients require close medical follow-up and strict treatment adherence, aiming to lower LDL-C as much as possible and reduce the residual atherothrombotic risk [14,15,16]. The aim of the present study is to describe patients’ adherence to PCSK9i during the COVID-19 pandemic.

## 2. Materials and Methods

### 2.1. Study Design and Study Population

Patients frequently undertaking PCSK9i therapy at the Cardiac Diagnostic Unit of the University of Campania “Luigi Vanvitelli”, and who missed the follow-up visit from 9 March 2020 and 17 May 2020, due to the COVID-19 pandemic lockdown restrictions, were retrospectively selected from the hospital healthcare database. Patients with missing telephone numbers, untraceable patients, and patients who did not give their informed consent were excluded.

### 2.2. Teleconsultation and Data Collection

Each patient underwent a semi-structured telephonic interview. The following clinical data were collected: comorbidities, recent medical history, pharmacological treatments, drugs adherence, and reasons for therapy discontinuation. All patients were also asked to send by dedicated email address or mobile number the blood laboratory tests they should have exhibited during the missed follow-up visit including total cholesterol, LDL-C, high-density lipoprotein cholesterol (HDL-C), and triglycerides. A CV risk profile was assigned to each patient, according to the most recent European guidelines on the management of dyslipidemias [13].

PCSK9i dispensation to patients before and during lockdown was checked on a dedicated digital platform made available by the Italian Medicines Agency (AIFA).

### 2.3. Primary and Secondary Outcomes

The primary outcome of the study was to evaluate the patients’ adherence to PCSK9i during the COVID-19 outbreak. The changes in lipid profile on treatment or after PCSK9i discontinuation were assessed as a secondary outcome.

### 2.4. Satistical Analysis

Kolmogorov–Smirnov and Shapiro–Wilk tests were used to evaluate the distribution of continuous data. Normally distributed variables were expressed as the mean ± standard deviation (SD), whereas non-normal distributed ones as the median and interquartile range (IQR). Categorical variables were reported as numbers and percentages. Continuous normally distributed variables were compared using the Student *t*-test; differences between non-normally distributed variables were tested with the Mann–Whitney U test. Categorical variables were compared with the chi-squared test, or Fisher exact test, when appropriate. For all tests, a *p*-value < 0.05 was considered statistically significant. Analyses were performed using R version 3.5.1 (R Foundation for Statistical Computing, Vienna, Austria). Patients’ adherence to treatment was measured through the medication possession ratio (MPR), dividing the total number of treatment days by the specific time of monitoring [17].

### 2.5. Ethics Approval

The study was conducted in accordance with the declaration of Helsinki and was approved by the local ethical committee (AOC-0035368-2020). Written informed consent was obtained from all study participants before data collection.

## 3. Results

**Figure 1:**
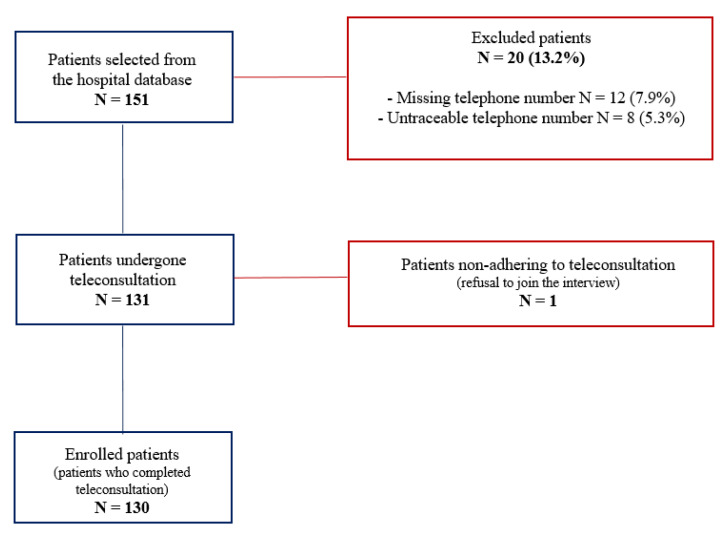
Inclusion graph of the study population.

There were 151 patients on PCSK9i therapy selected for inclusion from the hospital healthcare database, with 12 patients (7.9%) excluded for missing, 8 (5.3%) for untraceable telephone numbers, and 1 patient (0.7%) for refusing to join the interview. Finally, a total of 130 patients (63.5 ± 9.3 years, 68.5% males) who completed the phone interview and sent their blood exams by email or message were included in the study (Figure 1).

All patients were taking Evolocumab 140 mg every 15 days; 15 patients (11.5%) for primary prevention, and 115 (88.5%) for secondary prevention. At the time of study entry, the mean duration of PCSK9i therapy was 11 ± 3.4 months. Evolocumab was taken in monotherapy by 15 (11.5%) out of 130 patients due to statin intolerance. In the other cases, it was taken in combination with statins and/or other lipid-lowering therapies. Concomitant drugs of the study population are shown in Appendix A, Table A1 and Table A2.

There were 11 out of 130 patients (8.5%) who temporarily interrupted the Evolocumab therapy for a mean time of 65 ± 1.5 days and were considered the non-adherent group. No other drugs were discontinued, including the non-vitamin K antagonist oral anticoagulants (NOACs).

The causes of PCSK9i discontinuation were a failure in drug’s prescription due to temporary interruption of the non-urgent outpatient visits (*n*: 7; 63.6%) and a failure in the drug’s withdrawal due to patients’ fear of becoming infected by leaving the house or taking public transport during COVID-19 (*n*: 4; 36.4%).

The pre-pandemic clinical characteristics of the overall population and the comparison between the adherent and non-adherent groups are reported in Table 1.

**Figure 2:**
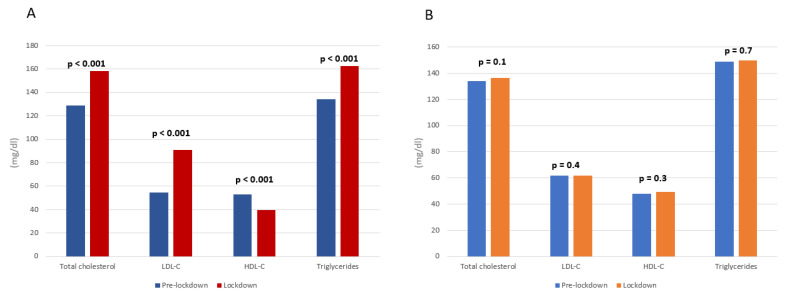
Lipid values changes between pre- and lockdown period in PCSK9i non-adherent (panel (**A**)) and adherent (panel (**B**)) group.

**Figure 3:**
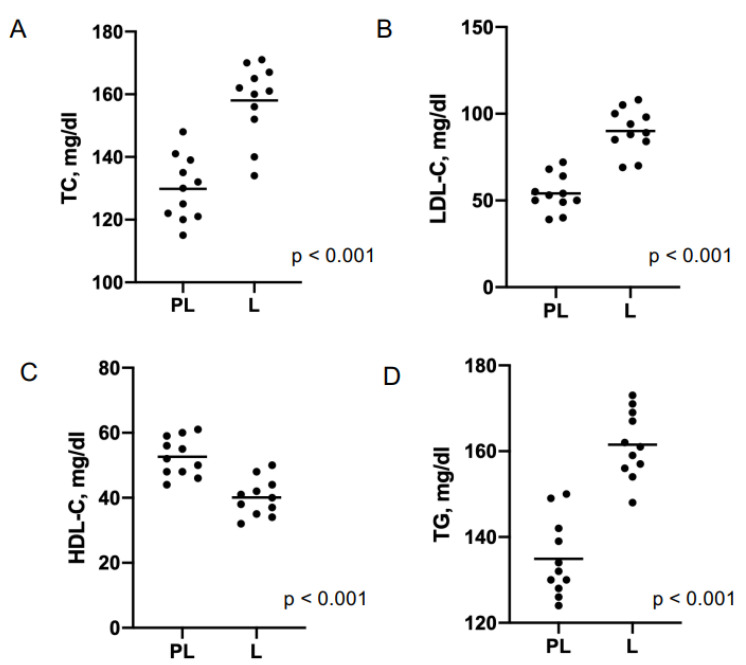
Scatter plots of pre-lockdown (PL) and lockdown (L) lipid values of non-adherent patients. TC: total cholesterol (panel (**A**)); LDL-C: LDL cholesterol (panel (**B**)); HDL-C: HDL cholesterol (panel (**C**)); TG: triglycerides (panel (**D**)).

Non-adherent patients were younger than adherents (61.3 vs. 68 years, *p* < 0.01) and showed higher prevalence of smokers (100 vs. 64.7%, *p* = 0.01), systemic arterial hypertension (54.5 vs. 15.1%, *p* < 0.01), use of beta-blockers (100 vs. 52.9%, *p* = 0.003), proton pump inhibitors (91 vs. 45.4%, *p* = 0.004), statins (91 vs. 75.6%, *p* < 0.001), and ezetimibe (100 vs. 56.3%, *p* = 0.005), and patients at very high risk (81.8% vs. 33.6%, *p* < 0.01). At follow-up, the non-adherent group showed a significant increase in total cholesterol (158.2 vs. 129 mg/dL, *p* < 0.001), LDL-C (90.8 vs. 54.4 mg/dL, *p* < 0.001) and triglycerides (162.8 vs. 134 mg/dL, *p* < 0.001) plasma levels, and a marked decrease in HDL-C plasma levels (39.8 vs. 52.8 mg/dL, *p* < 0.001), compared to pre-pandemic values (Figure 2 and Figure 3).

No changes in lipid profile were observed in the adherent group. The lipid blood values of both groups before the pandemic and during the lockdown are shown in Table 2.

At baseline, all of the 11 patients of the non-adherent group were in LDL-C therapeutic range, according to their CV risk. During follow-up, 9 patients (82%) did not reach the target anymore (*p* = 0.0002). Regarding the adherent group, no significant difference in LDL-C target loss was found during follow-up (7 vs. 9 patients, *p* = 0.797).

## 4. Discussion

The main findings emerging from this study include that 8.5% of patients on PCSK9i were non-adherent to therapy during COVID-19 lockdown. The leading causes of non-adherence to PCSK9i were failure in drug’s prescription due to temporary interruption of the non-urgent outpatient visits and failure in drug’s withdrawal due to patients’ fear of becoming infected during the pandemic. The non-adherent population showed an increase in LDL-C of about 67%; 82% of non-adherent patients moved out of the LDL-C therapeutic range during the lockdown.

It is well known that LDL-C is a causal factor for atherosclerosis; in particular, the effect of LDL-C on the risk of atherosclerotic CV disease (ASCVD) is determined by both the absolute magnitude and the total duration of exposure to LDL-C [18]. Lowering LDL-C blood values reduces the risk of ASCVD proportionally to the absolute achieved reduction in LDL-C [18,19]. The current European Guidelines for the management of dyslipidemias [13] recommend >50% reduction in LDL-C from the baseline in patients at high and very high CV risk, with strict treatment goals for LDL-C absolute values (<70 mg/dL and <55 mg/dL, respectively).

PCSK9i are effective and safe lipid-lowering drugs, recommended for secondary or primary prevention in patients at high CV risk not achieving their LDL-C goal on a maximum tolerated dose of statin and ezetimibe [13]. The commercially available PCSK9i, Alirocumab and Evolocumab, have been shown to significantly reduce LDL-C levels on average by 60% if administered alone and by 85% if administered with high-intensity statin and ezetimibe. More importantly, both proved to reduce the risk of CV events [9,10]. Moreover, PCSK9i demonstrated a higher level of adherence than statins, likely due to their favorable administration regimen, low rates of adverse effects, the opportunity to use a lower (more tolerable) statin dosage, and the patient’s perception that injecting therapy is more effective than oral [20]. Despite this evidence and the European guidelines recommendations, the rate of prescription of PCSK9 inhibitors appears below expectations, mainly due to national rules governing their reimbursement.

Moreover, during the COVID-19 outbreak, reduced adherence to PCSK9i therapy was observed, with a subsequent increase in LDL-C blood values of high CV risk patients. This phenomenon may be due to the strict limitations by the AIFA for national pharmacovigilance purposes concerning PCSK9i use [21]. Currently, only a few physicians are authorized to prescribe these drugs in Italy.

Moreover, the PCSK9i’s distribution occurs exclusively through the hospital pharmacies of the Local Health Authorities. Finally, the hospital pharmacist has to promptly communicate the occurred PCSK9i’s dispensation to AIFA in order to allow the following drug prescriptions.

This complex mechanism creates a PCSK9i access barrier, especially during a national health emergency, such as the COVID-19 pandemic.

Simplifying patients’ access to PCSK9i can be a strategy to reduce the risk of treatment discontinuation and ensure continuity of care. This goal could be achieved by increasing the number of physicians authorized for PCSK9i prescription and by involving several private territorial pharmacies in the drug’s distribution system.

Telemedicine proved to be a simple and well-tolerated tool for ensuring continuity of care and the outpatient management of patients with CV diseases during the COVID-19 pandemic [22,23]. Many telecommunication tools (e.g., live video conferencing, phone calls, remote monitoring of implanted or wearable devices) have been used to collect diagnostic and prognostic data when an in-hospital follow-up visit was not performed [22,24]. The use of telemedicine is of pivotal importance during public health emergencies such as COVID-19, where social distancing is mandatory, permitting patients to avoid direct physical contact, diminishing exposure to respiratory secretions, and minimizing the risk of virus transmission [25]. Its role becomes crucial in the clinical management of chronic diseases, such as CV diseases, which require many follow-up visits, potentially increasing the risk of infection.

Based on these considerations, it would be desirable to officially recognize the telemedicine service, addressing the concerns related to reimbursement policies and licensing laws in Italy. Furthermore, specialized professionals should be trained and educated as interviewers to ensure clear and effective communication with patients and successful data collection. Moreover, the activation of a national program for the digital literacy of the elderly is needed to increase adherence to telemedicine services.

## 5. Conclusions

The COVID-19 lockdown caused a remarkable lack of adherence to PCSK9i therapy, risking negative implications for the health status of high-risk patients with chronic CV diseases. Some strategies may be proposed to ensure continuity of care and the appropriate management of patients with high residual atherothrombotic risk, such as removing the barriers to PCSK9i prescription and patient access and enhancing the telemedicine services.

## Figures and Tables

**Table 1:** Pre-lockdown, clinical characteristics of the overall study population, and comparison between adherent and non-adherent groups.

Variables	Overall Population (*n* = 130)	Adherent Group (*n* = 119)	Non-Adherent Group (*n* = 11)	*p*-Value
Age (years), mean ± SD	63.5 ± 9.3	68 ± 4.5	61.3 ± 5.9	<0.001
Male, *n* (%)	89 (68.5)	84 (70.6)	6 (54.5)	0.3
Smokers, *n* (%)	24 (18.5)	77 (64.7)	11(100)	0.01
Hypertension, *n* (%)	88 (67.7)	18 (15.1)	6 (54.5)	<0.001
Obesity, *n* (%)	4 (3.1)	3 (2.5)	1 (9.1)	0.2
Familial hypercholesterolemia, *n* (%)	15 (11.5)	14 (11.8)	1 (9.1)	0.8
Diabetes mellitus type 2, *n* (%)	21 (16.1)	1 (0.8)	0 (0)	0.8
Coronary artery disease, *n* (%)	102 (78.5)	93 (78.1)	9 (81.8)	0.8
Stroke, *n* (%)	1 (0.8)	20 (16.8)	1 (9.1)	0.5
Peripheral arterial disease, *n* (%)	35 (26.9)	34 (28.6)	1 (9.1)	0.2
Atrial fibrillation, *n* (%)	2 (1.5)	2 (1.7)	0 (0)	0.7
Carotid atherosclerosis, *n* (%)	10 (7.7)	9 (7.6)	1 (9.1)	0.8
Chronic obstructive pulmonary disease, *n* (%)	4 (3.1)	3 (2.5)	1 (9.1)	0.2
High risk, *n* (%)	53 (40.8)	51 (42.8)	2 (18.1)	0.11
Very high risk, *n* (%)	49 (37.7)	40 (33.6)	9 (81.8)	<0.001
Moderate risk, *n* (%)	28 (21.5)	28 (23.5)	0	0.07
Patients on LDL-C target, *n* (%)	123 (94.6)	112 (94.1)	11 (100)	0.4
Beta blockers, *n* (%)	74 (56.9)	63 (52.9)	11 (100)	0.003
Antiplatelet drugs, *n* (%)	90 (69.2)	80 (67.2)	10 (90.9)	0.1
ACE inhibitors, *n* (%)	41 (31.5)	38 (31.9)	3 (27.3)	0.7
Angiotensin II receptor blockers, *n* (%)	33 (25.4)	32 (26.9)	1 (9.1)	0.2
Calcium channel blockers, *n* (%)	15 (11.5)	14 (11.8)	1 (9.1)	0.8
Diuretics, *n* (%)	43 (33.1)	37 (31.1)	6 (54.5)	0.1
Non-vitamin K antagonist oral anticoagulants *n* (%)	6 (4.6)	5 (4.2)	1 (9.1)	0.5
Class III antiarrhythmics, *n* (%)	6 (4.6)	6 (5.04)	0 (0)	0.4
Insulin, *n* (%)	6 (4.6)	6 (5.04)	0 (0)	0.4
Alpha blockers, *n* (%)	5 (3.8)	5 (4.2)	0 (0)	0.9
Antianginal drugs, *n* (%)	7 (5.4)	6 (5.04)	1 (9.1)	0.6
Antigout drugs, *n* (%)	5 (3.8)	5 (4.2)	0 (0)	0.5
Glucocorticoids, *n* (%)	1 (0.8)	1(0.8)	0 (0)	0.8
Levothyroxine, *n* (%)	6 (4.6)	6 (5.04)	0 (0)	0.4
Heparin, *n* (%)	1 (0.8)	1 (0.8)	0 (0)	0.8
Antiepileptic drugs, *n* (%)	1 (0.8)	1 (0.8)	0 (0)	0.8
Proton pump inhibitors, *n* (%)	64 (49.2)	54 (45.4)	10 (91)	0.004
Statins, *n* (%)	100 (76.9)	90 (75.6)	10 (91)	<0.001
Ezetimibe, *n* (%)	78 (60)	67 (56.3)	11 (100)	0.005

**Table 2:** Changes in blood lipid values in PCSK9i adherent and non-adherent patients.

	Lipid, mg/dL	Pre-Lockdown	Lockdown	*p*-Value
**Adherent group** **(*n* = 119)**	Total cholesterol	134 ± 12	136.5 ± 14	0.1
	LDL-cholesterol	61.6. ± 3.2	62 ± 3.6	0.4
	HDL-cholesterol	47.8 ± 12.1	49.3 ± 12.4	0.3
	Triglycerides	149 ± 21.9	150 ± 22	0.7
**Non-adherent group** **(*n* = 11)**	Total cholesterol	129 ± 9.8	158.2 ± 11.4	<0.0001
	LDL-cholesterol	54.4 ± 7.7	90.8 ± 6.02	<0.0001
	HDL-cholesterol	52.8 ± 5	39.8 ± 4.7	<0.0001
	Triglycerides	134 ± 8.8	162.3 ± 7	<0.0001

## Data Availability

The data presented in this study are available on request from the corresponding author.

## Appendix A

**Table A1:** Concomitant lipid lowering therapies.

Concomitant Lipid Lowering Drugs	N (%)
Ezetimibe 10 mg, *n* (%)	15 (11.5)
Atorvastatin 40 mg, *n* (%)	14 (10.8)
Rosuvastatin 40 mg, *n* (%)	6 (4.6)
Rosuvastatin 20 mg, *n* (%)	15 (11.5)
Rosuvastatin 10 mg, *n* (%)	2 (1.5)
Atorvastatin 20 mg + Ezetimibe 10 mg, *n* (%)	10 (7.7)
Atorvastatin 40 mg + Ezetimibe 10 mg, *n* (%)	20 (15.4)
Simvastatin 20 mg + Ezetimibe 10 mg, *n* (%)	8 (6.2)
Simvastatin 40 mg + Ezetimibe 10 mg, *n* (%)	12 (9.2)
Rosuvastatin 10 mg + Ezetimibe 10 mg, *n* (%)	5 (3.8)
Rosuvastatin 20 mg + Ezetimibe 10 mg, *n* (%)	8 (6.2)

**Table A2:** Concomitant non-lipid lowering therapies.

Concomitant Drugs	N (%)
Beta-blockers	
- Bisoprolol	53 (40.8)
- Nebivolol	7 (5.4)
- Metoprolol	6 (4.6)
- Carvedilol	5 (3.8)
- Atenolol	3 (2.3)
Antiplatelet agents	
- Acetylsalicylic acid	49 (37.7)
- Clopidogrel	9 (6.9)
- Ticagrerol	13 (10)
- Clopidogrel + acetylsalicylic acid	19 (14.6)
ACE- inhibitors	
- Ramipril	30 (23.1)
- Lisinopril	6 (4.6)
- Enalapril	5 (3.8)
Angiotensin II receptor-blockers	
- Losartan	9 (6.9)
- Olmesartan	11 (8.5)
- Telmisartan	7 (5.4)
- Valsartan	6 (4.6)
Calcium channel-blockers	
- Amlodipine	14 (10.8)
- Lercandipine	1 (0.8)
Diuretics	
- Thiazide diuretics	17 (13)
- Loop diuretics	14 (10.8)
- Potassium sparing diuretics	2 (1.5)
- Carbonic anhydrase inhibitors	1 (0.8)
- Osmotic diuretics	9 (6.9)
Non-vitamin K oral anticoagulants	
- Apixaban	3 (2.3)
- Rivaroxaban	2 (1.5)
- Dabigatran	1 (0.8)
Class III antiarrhythmics	
- Amiodarone	6 (4.6)
Oral antidiabetics	
- Biguanide	8 (6.1)
- Sulfonylureas	2 (1.5)
Insulin	6 (4.6)
Alpha-blockers	
- Doxazosine	5 (3.8)
Antianginal agents	
- Ranolazine	7 (5.4)
Antigout drugs	
- Febuxostat	5 (3.8)
Glucocorticoids	
- Prednisolone	1 (0.8)
Levothyroxine	6 (4.6)
Heparin	1 (0.8)
Antiepileptic drugs	1 (0.8)
Proton pump inhibitors	
- Omeprazole	14 (10.8)
- Pantoprazole	44 (33.8)
- Esomeprazole	3 (2.3)
- Lansoprazole	1 (0.8)
- Rabeprazole	2 (1.5)
